# NIH initiative to balance sex of animals in preclinical studies: generative questions to guide policy, implementation, and metrics

**DOI:** 10.1186/s13293-014-0015-5

**Published:** 2014-10-03

**Authors:** Louise D McCullough, Geert J de Vries, Virginia M Miller, Jill B Becker, Kathryn Sandberg, Margaret M McCarthy

**Affiliations:** 1 Department of Neurology and Neuroscience, The University of Connecticut Health Center, Farmington 06030, CT, USA; 2 Neuroscience Institute, Georgia State University, Atlanta 30302, GA, USA; 3 Department of Surgery, Mayo Clinic, 200 First St. SW, Rochester 55905, MN, USA; 4 Department of Physiology and Biomedical Engineering, Mayo Clinic, 200 First St. SW, Rochester 55905, MN, USA; 5 Department of Psychology and Molecular and Behavioral Neuroscience Institute, University of Michigan, Ann Arbor 48109, MI, USA; 6 Center for the study of Sex Differences in Health, Aging and Disease, Georgetown University, Washington 20057, DC, USA; 7 Department of Pharmacology and Program in Neuroscience, University of Maryland School of Medicine, Baltimore 21201, MD, USA

**Keywords:** Gender, Sexual dimorphism, Sex differences

## Abstract

In May of 2014, the NIH Director together with the Director of the Office of Research on Women’s Health announced plans to take a multi-dimensional approach to address the over reliance on male cells and animals in preclinical research. The NIH is engaging the scientific community in the development of policies to improve the sex balance in research. The present, past, and future presidents of the Organization for the Study of Sex Differences, in order to encourage thoughtful discussion among scientists, pose a series of questions to generate ideas in three areas: 1. research strategies, 2. educational strategies, and 3. strategies to monitor effectiveness of policies to improve the sex balance in research. By promoting discussion within the scientific community, a consensus will evolve that will move science forward in a productive and effective manner.

## 1 Review

### 1.1 Introduction

In May 2014, almost 20 years after the passing of the NIH Revitalization Act requiring inclusion of women in clinical research, NIH Director Francis S. Collins together with Janine A. Clayton, Director of the Office of Research on Women’s Health, announced NIH’s decision to address the over-reliance on male cells and animals in preclinical research [1]. This proposal arose from the realization that translating male sex-biased preclinical research to improvements in human health could result in adverse consequences for women’s health and that taking sex into account as a biological variable could improve reproducibility of research results [2]-[5]. The agency plans to take a multi-dimensional approach, which will include development of new policies, with oversight by the extramural research program [1]. One of the goals of these plans is to increase the use of female cells and animals in preclinical studies, thereby expanding the pool of data derived from females. NIH is engaging the scientific community in the development of these policies. The purpose of this commentary is to stimulate a thoughtful discussion among scientists about this issue, first, by posing a series of questions to generate ideas of various strategies the NIH could use to improve the sex balance in research, second, by suggesting ways to educate scientists on concepts of sex and gender and ways to implement those concepts into experimental design and, third, by discussing mechanisms to monitor the effectiveness of these strategies. It will be important that any new policy maximizes limited resources without adding burdensome regulations that hinder innovation. We note that there is no one “right” answer to any of these questions and that even the authors of this commentary at times disagree on the best strategies and approaches to recommend. However, we all agree that promoting discussion within the scientific community will ultimately evolve to a consensus that moves science forward in a productive and effective manner.

### 1.2 Rationale upon which to base policy: equity without equality

One way the NIH proposes to address the over-reliance on male cells and animals in preclinical research is to require grant applicants “to report their plans for the balance of male and female cells and animals in preclinical studies in all future applications, unless sex-specific inclusion is unwarranted, based on rigorously defined exceptions [1]”.

#### 1.2.1 Question #1: what would be reasonable exclusion criteria or “defined exceptions?”

##### Discussion

The answer is self-evident if one studies sex-specific conditions like preeclampsia, which is a condition of fulminating hypertension in pregnant women, or function and diseases in organs present only in one sex like the prostate and testes in men or the uterus and ovaries in women. However, if one investigates a condition that predominantly affects one sex, like breast cancer, which is 100× more common in women than men, does it make sense to include male animals or would this also be considered a “rigorously defined exception?” If it is an exception, would systemic lupus erythematosus, which afflicts women 10:1 offer another exception? Or are these two examples of where including males may identify mechanisms that may be targets for new therapies to reduce incidence or severity of these diseases in both women and men? However, if these criteria do constitute “exceptions”, then at what point would we determine that sex imbalance in disease incidence would no longer constitute reasonable grounds for exclusion of both sexes in preclinical studies? And, is the sex ratio of disease incidence the appropriate metric, or should it be based on the sex ratio of disease severity, age of onset, and/or outcome? Some diseases that occur less frequently in one sex than another exhibit a worse outcome in the under-represented sex, for example breast cancer in men, but is it because it may go undiagnosed until a later stage or because there is biological basis for a poor outcome?

##### Implementation

Applications could require that the investigator indicate if only one sex is being studied. If this is the case, the reviewers could more carefully examine the research design to determine if this is appropriate to the experimental design.

#### 1.2.2 Question #2: who would determine that the sex-specific inclusion criteria cited was unwarranted?

##### Discussion

Could an NIH staff member exclude an application before it reached the peer review panel? Or would this decision be left to a peer review panel with members that may or may not interpret the exclusion criteria the way they were intended by NIH? If the panel is not responsible for making this decision, would it fall to programmatic review? If so, how would disputes over this issue be resolved among principal investigators, peer review panels, and program officers? Currently, once a grant award is made, the principal investigator can modify the direction but not the goal of the research to follow their serendipitous discoveries to the benefit of biomedical research.

##### Implementation

A strategy to increase the use of females in experiments which requires investigators to present their plans for balancing male and female animals and cells will be successful only if the experiments are actually conducted and outcomes monitored.

#### 1.2.3 Question #3: is the optimal approach to achieve equitable sex balance in preclinical studies and in translating preclinical research to improving human health through policy change? If not, then what are the alternatives?

##### Discussion

Another major goal of the new NIH policy is to advance the translation of preclinical research to improve men’s and women’s health. Some in the scientific community say requiring investigators to study both sexes in preclinical research would waste precious research dollars. They argue that while many sex differences in biology exist, most drugs are similarly effective in men and women [6]. These observations suggest that physiological and pharmacological mechanisms are shared between males and females. Thus, conducting experiments in both sexes may often result in unnecessary duplication of data or data that provide limited added benefit to our current state of knowledge. The doubling of cells and animals will increase the costs not only of supplies but also of personnel time and will slow down progress due to the added workload.

Furthermore, some sex differences are species-specific and irrelevant to the human condition. For example, *Drosophila* represents a good species for studying some basic biological phenomena but do not model vertebrate sex differences. Other examples include hermaphroditic species and birds, which have either no, or distinctly different, sex chromosome complement than mammalian species.

On the other hand, an argument is made that we need to understand the physiology and pathophysiology of the female in the same detail as that of the male in order to avoid costs associated with withdrawing drugs from the market due to unforeseen adverse side effects in women. It cannot be known *a priori* that drugs and even drugs that are apparently efficacious in both females and males are utilizing the same mechanisms or should be administered in the same dosages [7]. Comparing disease causes and treatments between the sexes in preclinical research will lead to discovering novel drug targets [8]. Thus, how can we afford not to study both sexes in a balanced way?

Who bears the responsibility for generating, maintaining, and reporting the emerging data, which provide the evidence upon which to develop sex-based diagnostic and treatment algorithms? Should the responsibility for balancing research on the sexes be at the level of the individual investigator or at the level of grant review panels (study section)? Would requiring individual investigators to provide a rationale for why they were choosing to study only one sex and not the other and making this rationale a scorable peer review criterion raise awareness in the individual and among scientific specialties? And if so, would this awareness naturally lead to changes in thinking that ultimately resulted in less reliance of one sex over the other in future experimental design?

##### Implementation

Some argue that requiring each investigator to compare the sexes for at least one major component of the proposed study is reasonable and would not be overly burdensome. For example, if a proposal focuses on mechanisms of hypertension, then at least one experiment should compare the blood pressure in males and females in the model of hypertension studied. Or if one is studying mechanisms of alcohol addiction, at least one experiment should be required to compare the level of addiction between males and females in the model investigated. Others are concerned that this minimal requirement will not go far enough at balancing our understanding of male and female physiology and pathophysiology. However, uncovering substantial sex differences should lead investigators and review panels to include and require balances of sexes in subsequent experiments.

Would an alternative approach to achieving sex balance in preclinical research at the level of the individual investigator be to achieve this balance in aggregate across the NIH research portfolio? In other words, the total NIH budget for preclinical research would be spent equitably on female and male cells and animals. If the premise is that equity is achieved in total at NIH, the individual investigator is not obligated to study both sexes, nor is each program within each institute obliged to achieve a 50:50 split of research dollars spent on female and male preclinical research. This model acknowledges that the mission of some programs focuses on diseases that are more prevalent in one sex than the other, and because of these sex differences in incidence, it is not unreasonable to hold a preclinical research portfolio that exhibits a bias toward the over-represented sex.

How could NIH achieve this equity in preclinical research overall? One answer proposed is that NIH could leverage its existing chartered advisory committees including the councils, which are required by law and are part of every NIH institute. These advisory committees could develop specific requests for application (RFAs) to address key areas where data are deficient and thus encourage investigators to venture into those areas. For example, there is a deficit of research conducted in female models of chronic kidney disease because most available models of kidney disease exhibit little pathology in females. An RFA on mechanisms of chronic kidney disease in females would thus improve the sex balance in renal disease research. This approach would help to assure that the “best science” is funded.

However, would this approach give too much power to program, and in so doing, erode the widely valued principal among scientists of investigator-initiated research?

This equity in aggregate approach is conceptually similar to Title IX, the federal civil rights law, which prohibits sex discrimination in education. As the current pool of investigators studying females is far smaller than those studying male animals and cells, would this “Title IX” approach to achieving a sex balance in preclinical research [9] result in less competitive basic science grants being funded at the expense of more competitive grants? Or does “the best science” reflect the interdependency of biomedical research in broadly related fields assuring that the need to move forward in one field does not end up limiting progress in another?

### 1.3 Education

Any multi-dimensional approach to increase the number of female animals and cells in pre-clinical research should also be accompanied by NIH-sponsored educational initiatives. *First and foremost*, scientists need to be educated on the use of the terms sex (biology: sex chromosomes, hormones) and gender (psychosocial factors defining male and female). Not all researchers need to become experts in sex and gender biology or take a course in Sex Differences 101. However, all researchers should be aware that sex can influence the outcome of their experiments and must be accounted for as a critical biological variable. A question such as “is a particular mechanism found in males the same as it is in females?” is not the same as asking *why* the mechanism may be the same or different, or *when* and *how* sexual differentiation may occur. The NIH can promote the concepts of sex as a biological variable and the importance of sex differences and partner with other government and private institutions to embed these concepts into undergraduate and graduate science and medical education. These educational efforts will ensure that future scientists, review panels, and program officers will not need specific training but instead consider sex and gender within experimental design as “given.” Several resources are available to assist researchers in considering sex and gender in the design of their experiments (see the succeeding lists). NIH should encourage the expansion of these types of materials to meet the needs of the next generation of researchers and facilitate making such educational material readily accessible, e.g., online courses, webinars, and seminars. An easily accessible module on how to track the estrous cycle should be part of this package although some resources for this topic in rodents are already in the public domain [10]. A searchable catalog of reported sex differences in cells, tissues, genes, regulatory pathways, and behaviors is being developed in the private sector but it is expensive to develop and maintain (see the succeeding list).

The professional resources are as follows:

1. NIH funded Specialized Centers of Research on Sex Differences

 Available at: http://orwh.od.nih.gov/interdisciplinary/scor/index.asp

2. Textbooks

 Legato M. Principles of Gender-Specific Medicine. Elsevier. 2011. 2nd ed.

 Oertelt-Prigione, Regitz-Zagrosek. Clinical Aspects of Gender Specific Medicine. Springer. 2012

 Schenk-Gustafsson K, DeCola PR, Pfaff SW, Plsetsky DS. Handbook of Clinical Gender Medicine. Karger. 2012

 Regitz-Zagrosek, V. ed. Sex and Gender Differences in Pharmacology. Springer-Verlag 2012

 Mattison, DR. ed. Clinical Pharmacology during Pregnancy. Elsevier, 2013

3. Web-based continuing medicine education courses

 NIH ORWH Sex and Gender Differences in Health and Behavior. Available at: http://sexandgendercourse.od.nih.gov

 NIH ORWH The Basic Science and the Biological Basis for Sex- and Gender-Related Differences. Available at: http://sexandgendercourse.od.nih.gov

 TTUHSC Laura W. Bush Institute for Women’s Health. “Y” Does “X” Make A Difference? Available at: www.laurabushinstitute.org

 Women’s Health Info Site: Sex and Gender Resource for Clinicians and Trainees. Available at: http://whepducom.blogspot.com

 Resource listing of various educational modalities to use in integration efforts. Available at: http://www.drexelmed.edu/Home/OtherPrograms/WomensHealthEducationProgram/Resources.aspx

4. Web-based research and educational resources

 Sex and Gender Women’s Health Collaborative. Available at: www.sgwhc.org

 Stanford University’s Gendered Innovations. Available at: http://genderedinnovations.stanford.edu

 Canadian Institutes of Health Research. What a Difference Sex and Gender Make in Health Research. Available at: http://www.cihr-irsc.gc.ca/e/44082.html

 NIH Office of Research on Women’s Health Available at: http://orwh.od.nih.gov/sexinscience/index.asp

5. Articles outlining experimental design methodology

 Becker JB, Arnold AP, Berkley KJ, Blaustein JD, Eckel LA, Hampson E et al. Strategies and Methods for Research on Sex Differences in Brain and Behavior. Endocrinology. 2005;146:1650–73

 Greenspan JD, Craft RM, LeResche L, Arendt-Nielsen L, Berkley KJ, Fillingim RB et al. Studying sex and gender differences in pain and analgesia: A consensus report. Pain. 2007;132:S26-S45

 Miller VM, Kaplan JR, Schork NJ, Ouyang P, Berga SL, Wenger NK et al. Strategies and Methods to Study Sex Differences in Cardiovascular Structure and Function: A Guide for Basic Scientists. Biol Sex Differ. 2011;2:14. doi:10.1186/2042-6410-2-14

 Shah K, McCormack CE, A BN. Do you know the sex of your cells? Am J Physiol Cell Physiol. 2014;306:C3-C18

 Ritz SA, Antle DM, Cote J, et al. First steps for integrating sex and gender considerations into basic experimental biomedical research. FASEB journal: official publication of the Federation of American Societies for Experimental Biology 2014;28:4–13

6. Professional membership organizations

 Organization for the Study of Sex Differences. Available at http://www.ossdweb.org

 International Society for Gender Medicine. Available at http://www.isogem.com

7. Journals

 Biology of Sex Differences. Available at http://www.bsd-journal.com

The tool box with suggestions to achieve sex balance in preclinical studies modified from Figure Three of reference [11] is as follows:

1. *Develop* your knowledge of sex and gender

 Know the difference between sex and gender

 Avoid using the terms “sex” and “gender” interchangeably

 Review the literature to determine if there are sex or gender disparities for the phenomenon of interest

2. *Report* and *discuss*

 Always report the sex of the cells, tissues, animals, or participants you are studying.

 If there are data on both sexes, evaluate as such and report the differences.

 Justify the use of only one sex and note the limitations of this approach.

 Discuss the implications of sex and gender in relationship to the results.

3. *Educate* others

 As a reviewer, ensure that grant proposals or manuscripts:

a. Identify the sex of the experimental material

b. Justify the use of sex of the material

c. Use the terms sex and gender appropriately.

 As a mentor and colleague, ask questions as to whether sex and gender might be relevant to their work?

*A second essential aspect of education* promoted by the NIH will be to help scientists overcome misconceptions about using female animals. For example, a recent meta-analysis found that in most studies, the *variability* in male and female animals was comparable regardless of the stage of the estrous cycle in females. A factor that added the greatest variability to results was group housing males and females [12]. Therefore, it will be important for researchers to understand that hormonal variability cannot be used as a justification for excluding female animals from preclinical studies. The hormonal cycle can be controlled for in the experimental design. This concept needs to be understood by individual investigators, members of study section review committees, and reviewers and editors for scientific publications to ensure grant applications and papers that do not include females because of the estrous cycle are appropriately critiqued.

*Third and equally important*, scientists need to understand how to report results of sex differences without bias. It is inaccurate to report a sex difference in response as “better,” “improved,” or “worse” because this implies that one sex is the norm while the other is the deviant. Rather, sex differences should be reported objectively as “greater,” “less,” “higher,” or “lower” in one sex than the other.

*A fourth consideration* is statistical power and the ability to reliably detect or reject a sex difference in a given parameter. This is not a simple matter of *p* values but instead requires an accurate assessment of population variance, with males and females included, in order to calculate effect size (Cohen’s *d*). If an effect size is small (0.2 or less), then even a statistically significant difference may not be worth pursuing. Conversely, if an effect size is moderate to large (0.5 or greater), then even marginally significant probabilities might be of high biological significance. In both cases, the critical variable is a sufficiently large sample size to allow for a reliable estimate of population variance. Individual investigators working in systems they are familiar with should be able to predict the sample size needed to achieve this goal. Thus, it would be unwise to formulate policies that require the comparison of the two sexes unless the comparison is sufficiently powered to allow an evaluation whether the two sexes are different.

If sex differences are found, then the researchers should discuss the implications of this observation with respect to their overall study. For example, the mechanism of action they studied may be relevant to only one sex. Furthermore, their findings could warrant future research designed to investigate *how* and *why* males and females differed in this parameter (i.e., studies of sex chromosomes, gonadal hormones, and their interactions). Investigators also should acknowledge that even if sex differences were not observed in the one component of a system measured, it is not accurate to conclude that all the other components of this system are identical in females and males because functional or behavioral outcomes that are similar in the two sexes may be mediated by different fundamental mechanisms or may differ over the lifespan [13].

*Lastly*, even simple questionnaires can have an impact. In 2009, the Canadian Institutes of Health Research (CIHR) instituted a portfolio policy that required researchers to address four questions related to sex and gender in all grant applications (see the succeeding list) [14]. Similar approaches have been developed by European agencies [15]. Such questions require investigators to consider their hypothesis in the context of human health and sex disparities. Thoughtful answers to such questions help to educate reviewers as to the significance of the proposed project, and program officers could use the information to better balance a portfolio of projects studying female animals and sex differences. Critical to the usefulness of this approach is to have the answers to such questions become scorable review criterion.

The questions in grant applications submitted to the Canadian Institutes of Research are as follows:

Questions

1. Are sex (biological) considerations taken into account in this study? (Y/N)

2. Are gender (socio-cultural) considerations taken into account in this study? (Y/N)

3. If YES, please describe how sex and/or gender considerations will be considered in your research design (maximum of 2,000 characters)

4. If NO, please explain why sex and/or gender are not applicable in your research design (maximum of 2,000 characters)

### 1.4 Metrics and evaluation

Effectiveness of the new initiatives and policies in achieving the desired goal should be measurable. How then should we measure increases in the knowledge base on the biology of female animals and cells, increases in reproducibility of scientific results, and reductions in disparities in health outcomes between men and women?

Requiring a check box, filling in a chart, or answers to questions may result in compliance to the form without substantial change in practice, especially if the compliance is without meaningful consequences. For example, having investigators complete tables of numbers of male and female animals to be used in experiments will only affect the goals if the experiments are actually conducted and the results are reported by sex.

Following the implementation of CIHR’s policy requiring investigators to address issues of sex and gender in their proposals, an audit of CIHR applications funded from 2010–2011 found an overall increase in the percentage of researchers responding affirmatively to how their studies accounted for sex and/or gender. However, the trend for accounting for sex as an important biological variable varied by discipline, by nature of the researchers (basic versus clinical), and by sex of the researchers [14]. These CIHR results can be used to inform development and target groups for educational initiatives implemented by NIH that are designed to educate scientists about sex as a biological variable. Moreover, since the audit included only funded applications, it is unclear how these issues were considered overall by the scientific community or how review committees considered answers to the questions when deciding funding priorities.

Some practical steps to facilitate evaluation of the success of the proposed policies would be to require including the sex of the experimental material in the titles and methods sections of all research articles, a policy already implemented by several scientific journals [16],[17]. This reporting should be a requirement for research performed with NIH funds and evaluated by study sections as part of the standard review procedure. This should include reporting sex differences in genetically modified species. Listing papers published with grant support could be accompanied by a field that would indicate sex of the experimental material, numbers of males and females, and results reported by sex. Such a system would track outcomes from proposed work and provide an accessible monitoring system. Progress reports could be evaluated based on whether results collected included both sexes as proposed and whether results are reported in published papers for males and females separately. However, study sections will need guidelines for evaluation of progress reports and proposals. These steps will make it possible to evaluate short-term progress toward the goal. The long-term success will result only from improved treatment guidelines and patient outcomes with reduction in health disparities between women and men.

## 2 Conclusions

### 2.1 The status quo is not a viable option

Numerous lines of evidence indicate that the current status quo is not addressing fundamental issues of sex differences that are evident in gene expression, regulation of intracellular pathways, and disparities in health outcomes. It is critical to stop the current lack of attention to the sex of animals and cells and the current bias toward male subjects. A 2001 Institute of Medicine report provided recommendations for, and identified barriers to, advancing the science of sex differences, presenting several opportunities to advance science and medicine (see the succeeding list) [18]. Scientists, educators, and publishers were, and still are, in a position to implement these recommendations without regulatory intervention. However, because of the inertia and reticence among these groups to consider and implement the Institute of Medicine recommendations, here, we are 15 years later with the potential for additional governmental regulatory burden on the scientific enterprise.

The summary of recommendations from the Institute of Medicine modified from the Executive Summary of the Institute of Medicine report “Exploring the Biological Contributions to Human Health: Does Sex Matter?” [18] is as follows:

1. Recommendations for research

 Promote research on sex at the cellular level

 Study sex differences from womb to tomb

 Monitor sex differences and similarities for all human diseases that affect both sexes

 Mine cross species information

 Investigate natural variation

2. Recommendations for overcoming barriers to progress

 Clarity in the use of the terms sex and gender

 Determine and disclose the sex and hormonal status of research material

 Make sex-specific data readily available and easily accessible

 Support additional research, including interdisciplinary research, on sex differences

 Conduct and construct longitudinal and clinical studies so that results can be analyzed by sex

 Work to eliminate discrimination based on sex differences

Thus, going forward, development of policies to address deficiencies in our knowledge of sex and gender in biological mechanisms should encompass these areas: 1) at the individual investigator level, appropriate *rationale* for inclusion and exclusion for sex and gender in grant applications, 2) at the programmatic level, *accountability for investigator rationale* as part of scientific scoring of projects and *equity* in portfolio management, 3) *education* of researchers, grant reviewers, program officers, and journal editors regarding evaluation and reporting of sex and gender research, and 4) establishing clear *measures* that will assess progress toward including sex and female animals as critical variables in experimental design which can be translated to improved health outcomes for women and men. Cost analyses for implementation need to be performed. Ease of participation and innovative educational approaches to maximize compliance with defined goals should be key drivers for program development. These efforts will require combined input and collaboration among policy makers, scientists, and professional organizations to transform how science is done.

## Competing interests

The authors declare that they have no competing interests.

## Authors’ contributions

LDM, GJdeV, VMM, JBB, KS, and MMM were involved in the conceptualization, writing and editing of the article. All authors read and approved the final manuscript.
